# Assessment of data demand for informed-decisions among health facility and department heads in public health facilities of Amhara Region, northwest Ethiopia

**DOI:** 10.1186/s12961-023-01006-5

**Published:** 2023-06-26

**Authors:** Moges Asressie Chanyalew, Mezgebu Yitayal, Asmamaw Atnafu, Binyam Tilahun

**Affiliations:** 1 Department of Health Informatics, Institute of Public Health, College of Medicine and Health Sciences, University of Gondar, P. O. Box: 196, Gondar, Ethiopia; 2 Amhara National Regional Sate Health Bureau, Bahir Dar, Ethiopia; 3 Department of Health Systems and Policy, Institute of Public Health, College of Medicine and Health Sciences, University of Gondar, Gondar, Ethiopia

**Keywords:** Perception, Data demand, Data use, Evidence-based decision-making, Facilities and Department Heads, Public Health Facilities

## Abstract

**Background:**

Evidence-based decision-making is a foundation of health information systems; however, routine health information is not mostly utilized by decision makers in the Amhara region. Therefore, this study aimed to explore the facility and department heads' perceptions towards the demand for and use of routine health information for decision making.

**Methods:**

A phenomenological qualitative study was done in eight districts of the Amhara region from June 10/2019 to July 30/2019. We obtained written informed consent and recruited 22 key informants purposively. The research team prepared a codebook, assigned codes to ideas, identified salient patterns, grouped similar ideas, and developed themes from the data. Thus, data were analyzed thematically using OpenCode software.

**Results:**

The study revealed that health workers collected many data, but little was demanded and utilized to inform decisions. The majority of respondents perceived that data were collected merely for reporting. Lack of skills in data management, analysis, interpretation, and use were the technical attributes. Individual attributes included low staff motivation, carelessness, and lack of value for data. Poor access to data, low support for Health Information System, limited space for archiving, and inadequate finance were related to organizational attributes. The contextual (social-political) factors also influenced the use of eHealth applications for improved data demand and use among health care providers.

**Conclusion:**

In this study, health workers collect routine health data merely for reporting, and they did not demand and use it mostly to inform decisions and solve problems. Technical, individual, organizational, and contextual attributes were contributors to low demand and use of routine health data. Thus, we recommend building the technical capacity of health workers, introducing motivation mechanisms and ensuring accountability systems for better data use.

## Background

Health Information System (HIS) is one of the World Health Organization (WHO)’s frameworks with the role to ensure production, analysis, and use of reliable data [1, 2]. Strengthening of health systems has become a top priority of many global and national health agendas as a way to improve health outcomes [3–5]. Thus, governments need to make the best use of their limited resources within a context of a high disease burden, a growing population, and insufficient health services based on the available evidence [6]. Evidence-based decision-making is the primary function of national health information systems and is vital to the effectiveness of the health system as a whole [7, 8].

Increased information use in policy and program decision-making stimulates greater demand for data that, in turn, leads to more information use [9]. However, the study conducted in Mexico revealed that only 52% of the respondents utilized routine information for performance monitoring [10]. It was also noted that the majority of the Ministries of Health in Low and Middle-Income Countries (LMIC) are rich in data but information poor [11]. In Ethiopia, the level of routine health information use was inconsistent from place to place [12–15]. It ranged from the highest 78.5% in the North Gondar zone [16] to the lowest 37% in Jimma zone [17].

Effective HIS is expected to increase legibility, reduce medical errors, shrink costs, and boost healthcare quality [18]. Several factors that promoted demand and use of routine health data for decision-making were reported [19–22]. Thus, significant human and financial resources have been invested worldwide to enhance the data collection process. Unfortunately, this information is often not used for informing policy and programmatic decisions [9, 23].

Limited understanding of the determinants of performance of Routine Health Information System (RHIS) [10], data collection burden, and a high perceived work burden and negative attitudes toward change were the most frequently identified individual barriers for Evidence-Informed Decision Making (EIDM) [22, 24, 25]. Inadequate use of information was also stemmed from organizational issues such as the lack of a culture of information-use, lack of trust in the data, and the inability of program and facility managers to analyze, interpret and use [20, 23]. Besides, socio-cultural, political, and economic challenges have also influenced the demand and use of data [22].

Evidence-based decision-making is enhanced by a sound demand for health information, the collection and analysis of health data, and making information available to decision-makers [26]. Hence, Ethiopia has been implementing the Information Revolution (IR) agenda since 2017 to enhance demand for and use of routine health data at each level of the health system. Following the inception of the IR, the Amhara Region Health Bureau (ARHB) with the Federal Ministry of Health (FMoH) have invested tremendous efforts since then [27]. Despite all these efforts made, previous research work revealed routine health information is less utilized to inform decisions by health workers and decision-makers in the health system of the Amhara region[16, 28, 29]. They were quantitative evidences that lacked adequate information to understand the demand towards routine health data for decision-making and its attributes. Thus, qualitative research with adequate information to inform program planners, policy-makers and practitioners, in this regard is required. Therefore, this study aimed to explore the demand for routine health data for informed decision-making among department and facility heads.

## Methods

### Study design and settings

We applied a phenomenological qualitative study design to unveil the real experiences and in-depth understanding of facility and department heads in the healthcare system on data demand and use for evidence-based decision-making [30]. This research method was employed because of its nature. It helped to uncover the real scenarios of the demand for routine health data and showed the actual practices in using data for decision-making. Among the other qualitative study designs, the phenomenological qualitative approach also supported delineating valid recommendation considering the contextual factors in which healthcare services are delivered. As a qualitative researchers the research team had no established a priory relationship with study participants. Participants were received information only on the purpose of the study that enabled us to take informed consents. They were not provided information about the personal goals and interest of the researchers.

Data were collected from June 10/2019 to July 30/2019, in eight selected districts of the Amhara region, located in the northwest of Ethiopia. It comprised of 15 zones, 183 districts, and 3973 kebeles (493 urban kebeles). As of the 2020 annual report, the total population of the region was 22,191,890. Besides, there are 865 health centers and 81 hospitals (60 primary hospitals, 13 general hospitals, and eight referral hospitals) that provided healthcare services in the region [31].

### Study population, sample size, and sampling procedures

Facility and department heads working in public health facilities in the region were the study population. The study recruited participants purposively (using heterogeneous sampling technique) by the virtue of their position. It included the facility or departments’ heads who had more than six months of experience in the organization and who are supposed to utilize routine health information for patient care and management. However, the study excluded human resources and finance departments because they were not expected to utilize routine health data for their day-to-day activity follow-up. Initially, we determined 18 KIIs based on the available resource and time we had. However, our preliminary investigation showed us information was not saturated and we recruited additional participant until all research question were answered. Thus, 4 study participants were interviewed and finally the research included 22 key informants from 11 health facilities in eight districts in the region. Thus, the study maintained information saturation by taking ample respondents.

### Data collection tool and procedure

The data collection tool was adapted from the MESURE Evaluation data demand and use assessment tool [32, 33]. The instrument intended to assess the perception of facility or department heads towards data demand and use. It contained 14 main questions with multiple probes that helped to guide the interview. The tool was piloted outside the study area (Debre Tabor and Enjibara districts) in seven participants to identify ambiguous words, ensure flow of ideas, and check the comprehensiveness of contents and domains related with the research question.

The research team was comprised of one PI with Master of Science, and three co-researchers with Ph.D. level of education. All were male and working as senior researchers in the University of Gondar. The overall data collection process was coordinated by the research team who had received qualitative data analysis training organized by the University of Gondar and had conducted qualitative researches in different areas prior to this study. The Principal Investigator (PI) together with trained data collectors conducted the interview.

Three teams of data collectors comprised of one interviewer and one note-taker were deployed in data collection. Moreover, data collectors received a two-day training before the data collection to maintain the data quality. Single interview was carried out and it took around one and a half an hour on average to respond to all questions and guiding probes. Data were collected face to face using a qualitative interview guide and recorded using an audio recorder. Besides, expanded field notes were taken simultaneously and included in the main analysis. The Principal Investigator (PI) involved in data collection by supervising the overall data collection process, organizing preliminary findings from the interview, and investigating the level of information saturation.

### Data analysis

The audio data were transcribed to Amharic verbatim and back-translated to English. The research team sent back the transcribed data to the study participants confirming that the information provided was right and stated appropriately as said. We used OpenCode software version 4.02 for analysis. Codes of data with descriptions were developed as part of the data analysis and textual data were coded by the PI. Thus, data were reduced and categorized to find concepts. We identified salient themes, recurring ideas or words, and patterns from the data. We searched words or phrases used frequently, meanings in words, and unexpected results. The data were analyzed thematically to build overarching themes. Participant quotations were used for reporting the findings that were supportive or show disagreement from the previous findings and theories. We have searched for disconfirming results that violated what is believed to be true in the health care system from the data. In the end, the authors identified possible and plausible explanations for the results [34, 35]. In the end the research team had requested feedback from participant on the findings through email and incorporated their insights.

## Results

**Fig. 1:**
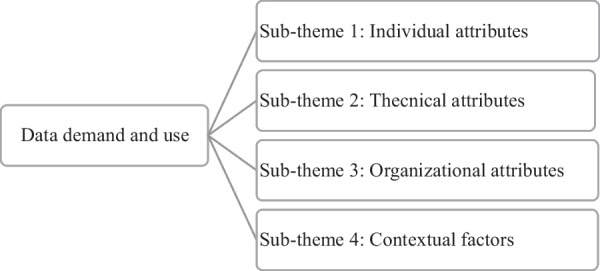
Pictorial presentation of main and sub themes

The study included 22 key informants in eight districts. Therefore, based on the information provided, one main theme (data demand and use) and four sub-themes (individual attributes, organization attributes, technical attributes, and contextual factors) have emerged, as shown in Fig. 1.

### Characteristics of the study participants

Of the total 22 Key-Informants (KI), 19(84%) were male participants. Besides, 11 were Bachelor of Science, seven were diploma, two were Master of Science, and two were Medical Doctors. The study participants' experience ranged from the highest 15 years to the lowest three years. The study participants comprised of Facility head office, Maternal and Child Health department, Expanded Program on Immunization department, Monitoring and Evaluation department, Antiretroviral Treatment focal, In-patient department, Out-patient department, and Chief Executive Officer (Table 1).

**Table 1:** Characteristics of study participants in Amhara region, northwest Ethiopia, 2021

Code	Position	Sex	Education	Profession	Experience in year
P1	Facility head	Male	Diploma	Clinical Nurse	5
P2	Delegate CEO	Male	MPH	Nutrition	10
P3	ART focal	Male	Diploma	Clinical Nurse	10
P4	EPI	Female	Diploma	Clinical Nurse	8
P5	CEO	Male	Diploma	Clinical Nurse	9
P6	MCH	Male	BSc	Nurse	6
P7	IPD head	Female	General Practitioner	Medicine	3
P8	OPD head	Male	General Practitioner	Medicine	4
P9	Facility head	Male	BSc	Laboratory	12
P10	Facility head	Male	BSc	Nurse	8
P11	Facility head	Male	BSc	Health Officer	5
P12	Pharmacy head	Male	BSc	Pharmacy	6
P13	CEO	Male	BSc	PHO	10
P14	OPD head	Male	BSc	Nurse	4
P15	Delegate facility head	Male	BSc	Nurse	5
P16	Delegate CEO	Male	BSc	Nurse	5
P17	Plan	Male	Clinical Nurse	Diploma	5
P18	ART focal	Female	Clinical Nurse	Diploma	12
P19	PMED head	Male	MHA	MSc	8
P20	Facility head	Male	BSc	HO	4
P21	Facility head	Male	BSc	HO	7
P22	HMIS head	Male	Diploma	HIT	15

*ART* antiretroviral therapy, *CEO* chief executive officer, *EPI* expanded program on immunization, *HMIS* health management information system, *IPD* in-patient department, *MCH* maternal and child health, *OPD* out-patient department, *PMED* planning, monitoring and evaluation department

### Data demand and use for decision-making

Participants decided on the program or administrative decisions while they were in the position they assigned. Program decisions were related to health service coverage, quality of care, performance achievement, data management, quality assurance, and supply management. Besides, human resource management, personnel administrations, social mobilization, renovation, and expansion of classrooms were among the administrative decisions they have made. Variant sources of evidence were utilized to inform decisions. The District Health Information System (DHIS) served as the main source of information to inform decisions. Besides, community scorecard, public opinion poll, Health Commodity Management Information System (HCMIS), client satisfaction survey results, and financial reports were alternative sources of information.

*We have obtained information from the facility's routine health information system and community scorecard to made decisions. In addition, we are using other data sources to monitor the implementation of the new reform in the facility. Thus, we considered the customer satisfaction survey result, too. [P11]*

Some of the participants perceived that the current information system supplied sufficient information to monitor performances and provide timely decisions; however, the majority of them explained that the current health information system had limitations in supplying adequate information on staff and client satisfaction and performance appraisals.

*We could not get data that indicated the client’s satisfaction concerning the services we provided them. Moreover, a balanced scorecard appraising the performance of health workers was not integrated into the routine health information system. As a result, performance appraisal was not done scientifically by comparing the result achieved to the targets set. [P15]*

Participants frequently disclosed that health professionals did not use health data to enhance patient care and management. Moreover, decisions were not made using the best available evidence and supported by data produced by the standard health information system..

*I do not want to hide the data use practice among the health workers in the facility. None of them have utilized information collected through routine HMIS for program follow-up or to ensure the quality of healthcare services. In this regard, we had challenges that we failed to resolve. [P20]*

Data sources for target setting, planning, and monitoring healthcare services were suggested by key informants. Routine health data, catchment population enumerations, financial and supply reports, and human resource reports served as sources of information. However, they explained research findings were not utilized for target setting or in decision-making. The FMoH and ARHB cascaded the strategic or annual plan targets to health facilities through Woreda-Based Planning; however, they did not compare these targets to survey results or other empirical evidence.

*We have used only the plan provided by the RHB. The catchment area population and the capacity of the hospital are not comparable. We only develop our plan by increasing 20% of the previous fiscal year instead of using the conversion factors as an alternative source of information. Therefore, we were unable to accommodate patient flow in the hospital. [P19]*

### Technical attributes

The technical attributes that affect producing trustworthy health data were noted by the study participants. The technical characteristics that were frequently noted were poor comprehension of data recording and reporting forms and poor data analysis and interpretation skills. Also, the DHIS2, a new HIS platform that the nation installed in healthcare facilities, could not be operated due to a lack of technical expertise among health professionals.

*We have no common understanding and knowledge in the data sources to compile reports, analyze indicators properly, provide possible explanations for the observed level of gaps, and identify the root cause for poor performance. [P5]*

Issues of data quality were a concern for many departments and facilities heads. They reported that data were fabricated in the facilities and reported to the next higher level. Data inconsistency between the source document and DHIS2 due to arithmetic and data encoding errors were also identified as common mistakes. Besides, weak data documentation, poor record-keeping, and delayed time in returning individual cards to medical record units were other technical attributes discouraging the demand for and use of routine health data for evidence-based decision-making.

*There was an occasion that data quality is a problem for us. Data were collected and reported by some health workers haphazardly without a thorough examination of all data sources [registers and tally sheets] to meet the due date only. Yes, I also observed that data recording and reporting tools were not utilized as per the guideline [P8]*

Quality data improves data demand and use. Thus, study participants mentioned strategies carried out in the health system to overcome the challenges of poor data quality. Data auditing and quality checks, Lot Quality Assurance Sampling (LQAS), Routine Data Quality Assurance (RDQA), and Data Quality Improvement Project (QIP) were some of the techniques applied to reduce data quality errors. Besides, the issues of data quality were discussed in the Performance Monitoring Team (PMT) meeting.

*We arranged an evaluation workshop at the district level to discuss and make clear the data quality issues with all HEWs and supervisors. They confessed that the data on hygiene and sanitation activities reported to the district office were false reports. …We have no system to make accountable those who send false data to the district office. Therefore, we decided to recount all hygiene and sanitation data and report to the next level. [P13]*

### Individual attributes

Respondents outlined multiple human-related factors that compromised the demand and use of information for decisions. Carelessness on the importance of data, low awareness for data ownership, unfavorable attitude towards data use, and low motivation were individual-level attributes. Besides, limited knowledge on gap identification, prioritization, root cause analysis, and action plan development among health workers hindered the culture of information use. Participants lacked value for data-driven decision-making, and they were not endeavoring to enhance the importance of informed decision-making at all levels.

*In my view, inadequate use of healthcare data for evidence-based decision-making was related to low awareness and limited capacity of health workers. It was considered in the facility that utilizing routine health data is the role of Health Informatics Technician (HIT) and facility administrators. [P3]*

### Organizational attributes

Health workers required data that have quality and trustworthiness to create an enhanced culture of data use. However, the majority of health facilities had maimed poor data management and rooms that were not spacious to accommodate source documents. Thus, they did not archive registers and tally sheets to maintain information for a long time. Besides, they did not create systems and strategies to hand over the source documents (Registers and tally sheets) to the next person. In line with this, planning and target setting in many health facilities were not based on previous years' data.

*It was not common practice to exchange all data source documents as other materials do. No one provided me all the data source documents that were used previously in this section. I found registers dumped in the store. Hence, I observed the facility had poor data management practice and archiving. [P11]*

Departments had prepared graphs or tables to present findings or share information with clients, service providers, and community representatives. Furthermore, monthly and quarterly meetings were organized and served as a means for information dissemination platforms. The majority of participants mentioned that a small budget was allocated for HIS activities, and they were not able to prepare broachers or leaflets. In addition, they lacked the knowledge and technical skill to develop regular information dissemination tools and annual magazines.

*We tried to display the main findings to clients and stakeholders within the organization, but we could not prepare broachers and leaflets to disseminate the information for wider circulation. We only provided feedback to case teams and lower-level structures to share good performances and weaknesses in the departments. [P8]*

The study showed that health facilities did not provide access to data for all health workers. They had limited access to the health information system to generate data and monitor their target achievement. Responsibility to access the databases in many facilities was given only to the HIT personnel.

*The data clerks deployed in the department was not volunteer to provide access to aggregate data to us. Even though I am responsible for the department, they were not enlisting to provide me access to the data. Thus, we were collecting the data by ourselves from the register and compared the performance against the target. [P18]*

### Contextual factors

Key informants mentioned that the social, political, and economic attributes positively impacted the operation platforms of the health information system. The advancement of Information Communication and Technology (ICT) drove the national government to develop the Information Revolution (IR) agenda and harness the Information ICT with the health system. The government moved forward to digitalize the HIS in the health systems and improved access to information for health workers, department heads, and facility heads.

*Yes. The DHIS2, for example, is the digital platform that supports access to information at different levels. Besides, the expansion of networking in the country also helped to create connected facilities in the district. The commitment from political leaders also contributed to draft the IR. [P19]*

Participants coined that digitalizing the HIS helped health workers getting patient information easily. The application of Electronic Medical Records (EMR) and DHIS2 in the health system enabled them to convert raw data into usable information. Besides, it supported the facilities to produce quality data that are sound to inform decisions and forecast supplies. Furthermore, it reduced the long reporting time from facilities to the national level, promoted the dissemination of information in a short time to audiences who are far away from the health facility, and enabled them to receive feedback from the community remotely.

*Political and economic environments influence the overall health information system. It helped to analyze data within very few minutes [computer-assisted data analysis], disseminate findings to other stakeholders, and find support from others. It also supported the community members and stakeholders to get information promptly. [P7]*

On the contrary, key informants mentioned that the political and economic environment influence the overall health information system negatively if utilized wrongly. Some health workers spent more time on social media by searching for news and information which are not relevant to improve patient care and management. Moreover, they abused the organization's website and pages posting unnecessary information that pushed health care seekers away from the facility.

*...Some health workers abused the website while they are posting unnecessary information about the organization. And the majority of health workers spent much time on social media searching for non-relevant information that created discomfort and dissatisfied patients and clients. [P18]*

## Discussion

The demand and use of information to inform decisions among facility and department heads were found to be limited. A low value for data, unfavorable attitude, and lack of motivation for routine health data were individual attributes. Besides, technical constraints like lack of skill in data analysis, visualization, interpretation, and dissemination affected the demand and use of data. Poor documentation, shortage of space to file medical records, limited access to information sources, and absence of recognition mechanisms for good performers in HIS activities were identified as organizational attributes.

The study evidenced that the routine health information was utilized inadequately, and demand for data to inform decisions was unsatisfactory. The finding was consistent with other studies done in Amhara Region [29], Southern Nations Nationalities and Peoples (SNNP) Region [36], and Eastern Ethiopia [12] that reported a low level of information use among health workers. It was also consistent with the study done in South Africa that mentioned inadequate use of information at district and facility levels to inform decisions and planning [23]. The low level of information use suggested that facilities were not monitoring health programs based on the available evidence effectively to attain the desired outcome with a reasonable cost [7]. However, the finding was inconsistent with the study done in North Gondar [16] and Hadya, SNNP [37], and Malawi [24] that reported high information use among health workers. It could be because of differences in study participants who were from districts with different achievements in RHIS activities, and for the Malawi study, it might be due to the nature of the intervention that was supported by partners.

Weak capacity and lack of skills to analyze, interpret and use data were the technical attributes. It was consistent with studies done in Kenya and Nigeria that reported weak human resource capacity, lack of skills to analyze, disseminate, and interpret data as perceived barriers that limit uptake and use of data for decision making [9, 22]. It was also in line with the study done in Malawi that highlighted poor data quality and collection burden constrained health data use at the district level [24]. It might be because of limited resource allocation in the region to enhance the healthcare providers' capacity in data analysis, interpretation, and use. Besides, the focus of leaders in the health system might not be as better as they gave to other activities. Thus, the limited capacity and lack of technical skills among key informants showed that information is collected merely for reporting and affects the program's effectiveness and efficiency.

A low value for data, unfavorable attitude, and lack of data ownership were individual attributes linked with low demand and health data use for decisions. The findings were consistent with the study done in LMIC, which reported participants had more data but did not utilize it because of a lack of ownership and positive attitude to the data [11]. Thus, low awareness and value for data affect the data demand and use for informed decisions. In turn, it fuels resource wastage that compromises the quality of health care in the region. However, the finding was inconsistent with the study done in Tanzania that reported good data ownership facilitated routine health data use [20]. It might be because of weak follow-up and limited financial support for HIS activities in the health system.

The organizational attributes were also accountable for enhanced data demand and use among health care providers. It was in line with the result obtained from East Welega that reported the organizational barriers as lack of accountability for the false report and low supportive supervision [37]. Besides, it was consistent with the findings obtained from Kenya that highlighted a lack of efforts to share information across organizations [9]. It might be because of a lack of attention to HIS activities and considering data management practice, accessing information to health workers, and information dissemination as simple tasks left to the HIT and other Planning, Monitoring, and Evaluation Directorate (PMED) personnel. Weak support from leaders and managers in the organization drives health workers to be demotivated to look for and use health data for informed decisions.

Contextual factors such as the social, political, and economic environment have influenced the application and utilization of eHealth solutions in the health system. It was in line with the study done in LMIC that outlined the effects of contextual factors in the health system [19]. It is a well-known fact that the financial constraint is higher in LMIC to operate the health information system [38]. Besides, the political commitment to available the required resources was not satisfactory. Thus, the finding showed that introducing advanced technologies and eHealth applications in the health system is highly influenced by the social and political environment.

## Implication of the study

This study highlighted the perception of facility and department heads who involved in a day-to-day decision-making process. It also revealed the technical, organizational, and individual attributes for demand and use of health data in the context of LMICs. Besides, it may serve as a source of information in designing interventions and enhancing the performance of the national HIS. It may inform policy and practice by providing insights about the real practice of data documentation and management on the ground.

## Limitations

We included department and facility heads but not for the health care providers that could forward additional insights. Participants might provide positive answers to questions so that social desirability bias might be introduced in the study. On the other hand, recall bias might also be inflicted because they replied to some questions by memorizing retrospectively.

## Conclusion

It was established that health professionals only collected regular health data for reporting purposes. The routine health information was not sufficiently demanded or correctly used to guide decisions and promptly resolve issues. They did not actively collect and make the necessary data for decisions available. Technical, individual, and organizational attributes have influenced the need and use of data among respondents. Besides, the social, political, and economic environment impacted the HIS-related activities that aimed to increase the demand or uptake of health data used for informed decisions. Hence, building the technical capacity of health workers in data management, analysis, interpretation, and use is required. Moreover, health workers at different levels shall be provided access to routine health data. Motivation mechanisms should also be in place to enhance the demand use of data for decisions. In the end, regulations and guiding principles should be enacted to enhance the wise use of internet services in the healthcare systems.

## Data Availability

Data will be available up on reasonable request from the corresponding author.

## Abbreviations

ARHB: Amhara Region Health Bureau
ARPHI: Amhara Region Public Health Institute
DHIS: District Health Information System
EMR: Electronic Medical Record
EIDM: Evidence-informed decision making
HCMIS: Health Commodities Management Information System
HIS: Health Information System
HIT: Health Informatics Technician
HMIS: Health Management Information System
ICT: Information Communication and Technology
IR: Information revolution
LMIC: Low and Middle Income Countries
LQAS: Lot quality Assurance Sampling
MOH: Ministry Of Health
PMED: Planning Monitoring and Evaluation Directorate
QIP: Quality Improvement Project
RDQA: Routine Data Quality Assurance
RHIS: Routine Health Information System
SNNP: South Nations Nationalities and Peoples
UoG: University of Gondar
